# “Oral health as an important milieu for social and mental health”: Perspectives of adolescents emerging from a qualitative study

**DOI:** 10.3389/froh.2022.879144

**Published:** 2022-08-18

**Authors:** Folake Barakat Lawal, Omotayo Francis Fagbule, Taiwo Akeem Lawal, Gbemisola Aderemi Oke

**Affiliations:** ^1^Department of Periodontology and Community Dentistry, University of Ibadan and University College Hospital, Ibadan, Nigeria; ^2^Fellow of the Consortium for Advanced Research Training in Africa, African Population and Health Research Center (APHRC), Nairobi, Kenya; ^3^Division of Paediatric Surgery, Department of Surgery, University of Ibadan and University College Hospital, Ibadan, Nigeria

**Keywords:** adolescents, mental health, oral health, school, social health

## Abstract

**Background:**

Recent evidence showed that the prevalence of oral diseases is still high among adolescents in many developing countries, including Nigeria. It therefore has becomes pertinent to focus on appropriate oral health interventions to promote oral health among them. This necessitates investigating the perspectives of adolescents, who are the major stakeholders, on the importance of oral health as baseline data needed in planning appropriate primary interventions.

**Aim:**

The aim of the study was to explore the perspectives of adolescents on the importance of oral health.

**Methods:**

An explorative qualitative study was conducted among adolescents attending senior secondary school I (grade 10) in the metropolis of Ibadan, Nigeria. A total of 12 focus group discussions were conducted among 120 adolescents aged 14 to 19 years. The discussions were transcribed verbatim, and triangulation and organization, as well as thematic analysis, of data were carried out.

**Results:**

Some adolescents had positive perceptions of oral health and valued it as highly important, although some had contrary views and believed oral health was unimportant. Oral health is described as being integral to general health, is associated with eating and communication, is a means of survival and confidence building, and is a prelude to attract the opposite gender. Adolescents perceived the need for oral health education and dental treatment.

**Conclusion:**

Adolescents have mixed perspectives on the importance of oral health; while some valued it as highly important, others believed it was of no significance. Those who valued oral health as important described it as a milieu for general health, human survival, and social and mental health and thus requested for oral health promotion in schools.

## Introduction

Over 3.5 billion people suffer from untreated dental problems globally, and disproportionately worse conditions are noted among those in low- and middle-income countries (LMICs), where inequality is more evident [1]. The two most common oral diseases globally, dental caries and periodontal disease, represent a high cause of unmet dental needs among adolescents in Nigeria [1, 2]. The prevalence of dental caries has been reported to be as low as 4.7%; however, the prevalence of untreated dental caries among the studied adolescents in Nigeria has been reported to be as high as 100% [3], while that of periodontal diseases was 73.3% [3]. Untreated dental conditions could further worsen the overall health of adolescents who are also at risk of other health issues, including mental, sexual, and reproductive health issues [4–7]. This is mainly because of the peculiarity of the adolescence period, which is a critical stage of human development where identities are established, self-awareness becomes prominent, and other relationships are being formed [8]. In addition, it is a stage where there is an eagerness to foster social interactions with peers, especially those of the opposite sex [8]. Thus, any instability in their daily activities or peer relationship because of oral health and other health issues could have consequential negative effects. Therefore, efforts should be geared toward promoting oral health among adolescents in Nigeria and other developing countries with similar conditions. In addition, the World Health Organization (WHO) and the World Dental Federation (FDI) had emphasized the need for promoting oral health to reduce the burden of oral disease among populations [9]. Furthermore, the connection between oral health and health conditions such as mental, sexual, and reproductive health among adolescents has led to the proposed integrated one-stop-shop approach in addressing their health issues [10]. This suggestion is also in line with the WHO's concept of having health-promoting schools, where students are exposed to health education and behavioral programs, which are aimed at enabling students to have control over the determinants of health and where their health issues can be promptly addressed holistically [11].

Unfortunately, this has not been achieved in many parts of the world, including Nigeria, and most Nigerian adolescents are not currently exposed to such effective, integrated health interventions [10]. This further supports the urgent need for appropriate interventions among adolescents in view of the importance of this period in the establishment of habits, which often continue into adulthood [8].

Planning of such programs requires baseline data on the self-perceived needs and importance as it relates to oral health and other health issues. This is crucial as the value of importance attached to oral health by adolescents, as well as the context in which its importance is viewed by them, is uncertain. In addition, the acceptability, utilization, and, ultimately, the success of any oral health intervention are connected with the level of importance that the beneficiaries (adolescents) attach to oral health. Hence, this study aimed to understand adolescents' perspectives on the importance of oral health and explore the relationship of their oral health status with other health and social issues.

## Materials and methods

An exploratory qualitative study design was used to explore the opinions of students about the importance of oral health and self-perceived oral health needs through focus group discussions (FGDs). The focus group discussion was chosen as the most appropriate design to obtain the required data from the adolescents because it allows discussants to generate ideas and challenge ideas by others through interactive discussions, thus making it easier to understand their views on the topic [12]. The study was conducted among adolescents attending randomly selected schools from four local government areas (LGAs) within the metropolis of Ibadan. Ethical approval for the study was obtained from the Oyo State Ethics Review Board (AD 13/479/743). Permission to conduct the study was obtained from the Oyo State Ministry of Education and from the principal of each selected school. A public school was randomly selected using a table of random numbers from each selected LGA to ensure that each LGA was included in the study. Students recruited for the study were purposively selected from one randomly selected senior secondary school I (grade 10) class in each school. The purpose and details of the study were explained to students in the selected class as a group. Thereafter, consent forms were distributed among the students who signified intention to participate in the study and who met the inclusion criteria to take the forms home to their parents. Only students who gave assent or consent depending on their age and with no special needs were included in the study. Students whose parents did not consent or who were ill at the time of the study were excluded.

The discussions were open and audiotaped involving 7–12 students per group. A minimum of two FGDs (two separate discussions per selected school based on gender) was conducted in each LGA to ensure robust data collection. The discussion was gender-based to avoid inhibition that may arise when adolescents of the opposite gender are mixed in the same group. After the daily FGD sessions, there was debriefing with a researcher (TA) [13]. This was to ensure that there was completeness of data to correct decision-making on the need to explore new information in subsequent interviews or not. The FGDs were continued until saturation was achieved when there was no new information obtained from the adolescents with consensus from the research assistants involved in the discussions and in the process of debriefing. A total of 12 FGDs were conducted among 120 students purposively selected from schools from four out of five local government areas within the metropolis of Ibadan.

A focus group guide was developed and used to guide the moderator during the discussions. The discussion questions were based on the literature review. The face validity of the focus group guide was assessed among adolescents in a school outside the four LGAs selected for the study, as was also ascertained by experts in the field of community dentistry. Thereafter, the focus group guide was fine-tuned and pretested in two groups of 12 students each from two schools in the LGA in Ibadan metropolis that was not selected for the study, to assess its comprehensiveness. It was found to be valid and comprehensive. The following questions were included in the focus group guide: “How do you feel when you saw the letter of invitation that the dentists will be coming to your school today?” “How important to you is the health of your teeth and mouth?” “Is taking the health of your teeth and mouth serious of any benefit?” “Do you perceive any oral health need?”.

The FGDs were conducted in a designated quiet classroom as provided by the schools. The discussions lasted between 25 and 30 min for each session. The discussions were moderated by one of the authors (FB) who had been trained in qualitative data collection, and the moderator was assisted by two trained research assistants. Also, one of the research assistants helped in taking notes of non-verbal expressions during the discussions. The second research assistant wrote the verbal responses, and these responses were compared with the tape-recorded discussions. Both assistants were trained in the facilitation of FGDs. During each discussion, probes were used to obtain more details from respondents. Also, paraphrasing of information provided by respondents was performed at intervals to confirm that information provided by the students was adequately and properly captured. In addition, a summary of key findings was given after each discussion. The discussions were reviewed after each session and ended after saturation, whereby no new emerging theme was noted. The saturation of data was determined by one of the authors (FB) in conjunction with the two research assistants. The respondents were asked to enter their sociodemographic details into a proforma prior to each discussion. The parent occupation was categorized as “skilled,” “unskilled,” and “dependents based on a modification of the Office of Population Censuses and Surveys (OPCS) that had been used in this environment [14].

### Trustworthiness of data

Based on the criteria of trustworthiness to ensure rigor and quality of qualitative data, four criteria have been identified: credibility, transferability, dependability, and confirmability [15].

### Credibility

To ensure credibility, the focus group guide was developed by the research team, which comprised public health dentists and epidemiologists with experience in qualitative research. The questions were developed through an extensive literature review of qualitative studies. In addition, the moderator of the discussions and the research assistants were trained in facilitating focus group discussions. The students were selected using the purposive sampling technique, and students from different socioeconomic classes were selected to ensure diversity of participants and robust data. Probes were used to ensure robust discussions and repetition of the responses of the adolescents, and a summary of the responses at each discussion was provided to ensure correct capturing of the responses of the adolescents. Also, the comparison of the written notes was taken by a research assistant during the FGDs with the audiotaped recordings. This was to minimize bias and ensure the correctness of data captured at the discussions.

### Transferability

The sociodemographic characteristics of the adolescents, details of the study design, focus group guide development and validity, and data collection and analysis have been provided for ease of placing the results in context, thereby aiding comparability of the finding with other studies.

### Dependability

The process of conducting the research has been well-described to enable replication by others.

### Confirmability

The adolescents who participated in this study were from senior secondary school 1 classes, thus forming a homogeneous population with the same experiences. In addition, data were collected from multiple regions of four LGAs within the city and from multiple schools within the LGAs to enhance triangulation of the data, enriching the robustness of the data with male and female students contributing equally to the population of the discussants.

Audiotapes were transcribed verbatim, transcripts were validated by two independent trained research assistants, and one of the authors compared the accuracy of the transcripts with recordings of the discussions on the audio tapes. Discrepancies noted were corrected after the discussion of the research assistants with one of the authors. Coding was carried out manually by one of the authors by reading through the transcripts systematically to identify themes, after which a review of the data was conducted together with the research team until saturation was reached when no new emerging themes were found, and this resulted finally in stoppage of data collection. Transcripts were coded based on these themes, and two independent researchers verified these codes by applying them to the transcripts to validate the process and ascertain reliability by triangulation [16]. The analyst triangulation was carried out in such a way that the two independent researchers who were epidemiologists applied the codes to the transcripts and these were compared [17]. Further analyses were conducted using NVivo version 12. The codes were grouped into categories, and an overarching theme was allotted. Further analysis of data was based on thematic induction. The data from the field observation of the participants' expressions were also summarized.

## Results

A total of 120 students participated in 12 FGDs. They were all in the 10th grade, and 60 (50.0%) were females or female adolescents. The mean age of the adolescents was 15.2 (±1.2) years (Table 1).

**Table 1:** Sociodemographic characteristics of the adolescents.

**Sociodemographic characteristics (*n* = 120)**	**Frequency (%)**
**Gender**	
Males or male adolescents	60 (50.0)
Females or female adolescents	60 (50.0)
**Parent's occupational class**	
Skilled	40 (33.3)
Unskilled	51 (42.5)
Dependent	29 (24.2)

The emerging themes were identified and grouped into three categories (Table 2): feelings toward oral health invite (excitement, apprehension, chastisement, and indifferent), the importance of oral health, and self-perceived oral health needs.

**Table 2:** Categories themes and subthemes emerging from the analysis.

**Categories**	**Themes/ Subthemes**
Feelings toward dentists	Excitement
	Apprehension
	Happiness
	Indifference
Importance and benefits of	**Need for survival**
the teeth and mouth	Eating
	**Social**
	Communication
	**Mental health**
	Confidence building
	Peer acceptance
	**Prelude to attract the opposite gender**
	Romantic advances
	Attractiveness
	**Integral to general health**
	Disease prevention
Self-perceived oral health	Knowledge of oral health
needs	Dental treatment needs
	School oral health program

*Bold names are themes and plain fonts are subthemes under each theme*.

### Feelings toward oral health invite

In the schools, there were mixed views on the letter of invitation about participating in an interview on oral health in schools; while there were positive views, some were negative and others indifferent. The participants expressed their views as positive; some were happy, excited, and anxious as they were eager to know more about the teeth and mouth on receiving the letter of invitation that they were to discuss about oral health. Some of the statements were as follows:

“*…and when I saw the form that it was from the dentist, I was happy that I would know more about the teeth.”*

“*I felt eager to know about the uhum pleasant organ that is the teeth. To know more about our teeth.”*

“*I felt delighted because I will be able to learn and gain more knowledge about our oral health.”*

“*I felt happy because I will be able to know some things about my oral health so that it will help me to improve me in areas where I am deficient.”*

“*I felt excited, and I was eager to know what the team had in stock for us.”*

“*…very very happy because…., we will have more information concerning oral health.”*

Some were apprehensive as they expressed their fears “*…, I thought that they wanted to come and remove our teeth but when I saw the posters and educational materials brought by the team, I realized that they came to teach us instead.”* (Female adolescent)

“*Some said you wanted to come and remove our teeth, so I thought so too and because of that, I did not want to come but when I got here and I saw that it was not so … I first looked closely at those inside before entering here.”* (Male adolescent)

“*I thought they wanted to remove my teeth and they will say that I should…….… I should be taking care eh, I should be taking care of my teeth every time.”* (Female adolescent)

Others were indifferent and undecided as mentioned by a student “*………. I felt happy and sad. I think that it is going to be helpful, boy, to see many dentists in our school.”* (Male adolescent)

While another student mentioned that “*…………. I do not feel anything, I do not know.”*

### Importance of oral health

The adolescents provided different descriptions of how they viewed the importance of oral health. The importance of the mouth was discussed around the needs for survival, social interaction, and mental health and as a prelude to attract opposite gender and look good, and it being integral to general health.

#### Survival

Some students ascribed the importance of oral health to eating and functionality and thus a means of survival.

“… *but let me just say that a person cannot survive because of what we can use our teeth to do.”* (Female adolescent)

“*The health of our teeth is so important because if there are no teeth, ah, we cannot eat... we cannot eat, that is the truth.”* … “*without the mouth, I cannot survive.”* (Male adolescent)

…* “Eh en, what I think, what I want to say is that if there are no teeth, we cannot eat, because it is important for us to have teeth so that we can eat. “… the importance of my teeth is that I like, I love food so without the teeth, I cannot eat.”* (Male adolescent)

#### Social interaction

Those who had recognized the importance of oral health described it as a must for social interaction.

“*It is very important to me because health is wealth.”*

“*It is very important because everywhere we go, we just have to express ourselves and communicate.… because ehnn we cannot do without communicating.”*

#### Prelude to attract opposite gender: Romantic advances

Oral health was described as a means of facilitating romantic advances with poor oral hygiene denoting failure.

“*It is so important to me because I know fully well-that I am a man, you know as a man I will have to toast girls …………… bad result when talking if the teeth are not clean.”* (Male adolescent)

Poor oral health was also described as a cause of unattractiveness.

“*… if you take something that damaged your teeth, your teeth will give you signs. It will start to shrink, it will start to decay, it will start to decay in a way that when your teeth are being presented outside it won't look attractive at all.”* (Female adolescent)

#### Mental health

Confidence in terms of social interaction and functional dentition was also mentioned as outcomes of good oral health.

“*… if you have clean teeth, you will be confident, and your mind will be at rest that you can eat anything.”* (Male adolescent)

The benefit of good oral health was linked with confidence as opined by the adolescents.

“*It is of benefit for me so that I can talk in the society very well as I like.”* (Male adolescent)

“*With clean teeth, you have confidence to talk anywhere, if you have mouth odor you will feel shy because you believe people will cover their mouth when you talk. So, it is good to have clean mouth.”* (Female adolescent)

Good oral health as a dictator of acceptability by peers.

“*Good oral health is very important because if your mouth is smelling, your peers and friends may avoid you. They will be saying ‘ah, this person, her mouth is smelling'. So, they will not be able to relate with you again.”* (Female adolescent)

Teeth lasting through life were also a reason for keeping the oral cavity healthy.

“*… so that you would be old with the teeth.”* (Male and Female adolescent)

#### Oral health was described as being integral to general health

The absence of oral and general health problems was also mentioned as the benefits of good oral health.

“*Yes, there are benefits. If we don't take care of our teeth, our mouth can be smelling and we can have tooth decay. By that time, we can contact a disease. So, there are benefits in taking care of our teeth.”* (Female adolescent)

“*… because disease can enter our body through any means…Yes, there are benefits so that bacteria ……….. so that we would not get sick or have sickness.”* (Male adolescent)

#### Oral health was described as non-important

A negative opinion was also iterated by a student.

“*… because everyday things change, I don't think oral health is beneficial to our personal growth.”* (Male adolescent)

Some described the teeth and mouth as of no importance without reasons. “*It is not important at all.” (*Male adolescent*)*

### Self-perceived oral health needs

The adolescents were positive about the need to have more information about oral self-care and dental care services.

“*I don't have enough information………. It is very important to know more about our teeth, how to keep it clean and how good it is to brush our teeth every day.”*

“*… by supplying us with essential things to brush our teeth, to enlighten us on how to brush our teeth. You should give us free dental treatment.”*

“*… by organizing a program separately for students to educate them on how they can take care of their teeth.”*

## Discussion

Findings from this study showed that the perspective of the students on the importance of oral health was mixed. While some believed it was important, others mentioned that it was not. The importance of oral health was built around the need for survival, social interaction, confidence building, acceptability by peers, enhancement of romantic advances, and disease prevention. This summarizes the importance of oral health as being integral to general health, a milieu for survival, mental stability, and social health, and a prelude to attract the opposite gender and look good.

The perspective of the students concerning the letter of invitation to discuss about oral health in school was mixed. While some were happy and excited that they would gain knowledge, others were apprehensive or indifferent. The happiness of students about gaining oral health knowledge has been described as a positive attitude toward oral health [18, 19]. On the other hand, some students were apprehensive, which could be linked to fear of tooth removal or self-perceived sub-optimal oral health that would require dental treatment as opined by adolescents in this study. Apprehension of adolescents caused by thoughts of dental treatment in the form of tooth extraction has similarly been reported by others [19, 20].

Adolescents considered oral health as an important means for survival. This revolved around eating and communicating with others. This finding is not surprising as eating was mentioned as a major importance of the oral cavity by adolescents in a previous study [21]. The role of diet in daily living is well-established. Furthermore, communication has been described as an important tool for positive peer relationships and adjustment to adolescent life [22–24]. The role of the oral cavity in human survival based on dietary requirements and communication as a major component of social interaction among adolescents is noteworthy in this study. This finding is consistent with that of other studies among adolescents [19, 20].

Confidence building, an attribute of self-esteem, mental stability, and social living among adolescents, was mentioned as a benefit of good oral health by the adolescents [5]. Lack of confidence and low self-esteem have been associated with poor adjustment modes, leading to depression and suicidal tendencies among adolescents [25]. Poor oral health, described as mouth odor and tooth decay, was mentioned by the adolescents to have consequences such as shyness of the affected person and unacceptability by peers. This is consistent with previous studies where having carious, missing, poorly arranged, and broken anterior teeth were found to affect adolescents' psychosocial behavior and self-esteem [6]. This was consequently linked to poor mental health among affected adolescents. In addition, peer rejection during adolescence has been associated with poor mental health, social incompetence, and maladjustment into adulthood [24]. The role of oral health in the development and maintenance of positive peer relationships with future advantages of mental stability, as well as life adjustment skills, needed for adolescents' growth and well-being in later life is indispensable. A need to prevent oral diseases and promote oral health among adolescents is thus essential and requires urgent attention if the proposed agenda of good health for all is to be achieved.

Good teeth appearance was associated with being attractive by the adolescents in this study. The appearance of the teeth, among other reasons, has similarly been mentioned as an essential part of oral health and a prerequisite for social life among adolescents in previous studies [18, 20]. Also, adolescents have a perception that having good teeth has a positive effect on their self-esteem [7]. In addition, perceived poor self-image by adolescents has been linked to poor mental adjustment and higher tendencies for suicidal thoughts and other conditions such as underachievement in school and risky behavior [25].

The role of oral health as a prelude to attract the opposite gender and look good was also mentioned by the adolescents. While female students stated that good oral health is an essential instrument for being attractive, male students reiterated that it serves as a means of romantic advances toward female counterparts. The adolescence period has been characterized by an eagerness to foster social interactions with peers, especially those of the opposite sex, and in forming relationships [8]. In addition, middle and late adolescence periods have been characterized by increased social interactions as the transition into adulthood is nearer [25].

Dental treatment was a major self-perceived oral health need mentioned by the adolescents. This may not be unexpected as high unmet dental needs among adolescents in the city have been reported [3, 26]. and may partly account for this. In addition, the financial implication of dental treatment and accessibility, among other factors, may also be responsible for the high desirability of dental care services in schools by adolescents. This could also explain the anxiety and fear exhibited by some students at the onset of the interviews that probably their oral health was sub-optimal, requiring treatment, which to them may be tooth extraction. Furthermore, they could be afraid that their oral health could be assessed as unfavorable by dentists and, if obvious to their peers, could have the consequence of rejection as an untoward effect, affecting mental health and schoolwork. The relationship between oral health problems and mental health challenges such as self-esteem, self confidence, self-competence, and self-rated dental health has been reported [4].

The adolescents also desired oral health education to know more about the teeth and self-oral care. This reflects the level of oral health knowledge in the populace, which had been described previously as sub-optimal in this environment [3, 21]. In addition, the students also had this premonition when they received the letter of invitation for the study as it is the major reason why many of them were happy on reading the information in the letter. The adolescents would also appreciate it if oral health education programs are organized for them separately, which indicates a form of desire for oral health intervention in schools.

Oral health was described as integral to general health by adolescents. In addition, the common usage of “health is wealth” was also supported in the discussions. This further corroborated the importance of oral health to adolescents and the need to promote oral health among them in Nigeria and other developing countries where there is a high burden of oral diseases. Moreover, it is important to consider extensive oral health promotion programs that take into consideration the determinants of health that could hamper the uptake of healthy oral health behavior among adolescents in schools so as to promote equities and not inequalities [11, 27].

In conclusion, this study showed that among adolescents, oral health creates a milieu for survival, social life, acceptability and peer relationships, prelude to attract opposite gender and to look good, which cannot be over-emphasized. Oral health could therefore be described as a premise for social growth and mental stability among adolescents, which, if optimal, may foster the emotional and social competencies of adolescents.

The limitation of this study is that it is purely qualitative, and thus, causality cannot be inferred from mental health, reproductive health, and oral health. However, it has the strength of having the baseline data needed to plan interventions. In addition, it confirms the previous speculation on the association between oral health and other health issues, such as social and mental health.

## Data availability statement

The raw data supporting the conclusions of this article will be made available by the authors, without undue reservation.

## Ethics statement

The studies involving human participants were reviewed and approved by Ministry of Health Oyo State Ethics Review Committee. Written informed consent to participate in this study was provided by the participants' legal guardian/next of kin.

## Author contributions

FL conceptualized the study, wrote the proposal, collected and analyzed data, and wrote substantial parts of the manuscript. OF assisted with data collection and contributed to the writing of the manuscript. TL contributed to the review of the proposal, design of instruments, data collection, and writing of the manuscript. GO supervised the drafting of the proposal and data collection and contributed to the writing of the manuscript. All authors approved the final version of the manuscript.

## Funding

This research was supported by the Consortium for Advanced Research Training in Africa (CARTA). CARTA is jointly led by the African Population and Health Research Center and the University of the Witwatersrand and also funded by the Carnegie Corporation of New York (Grant No. G-19-57145), Sida (Grant No. 54100113), Uppsala Monitoring Center, Norwegian Agency for Development Cooperation (Norad), the Wellcome Trust (reference no. 107768/Z/15/Z), and the UK Foreign, Commonwealth and Development Office, with support from the Developing Excellence in Leadership, Training and Science in Africa (DELTAS Africa) Programme.

## Conflict of interest

The authors declare that the research was conducted in the absence of any commercial or financial relationships that could be construed as a potential conflict of interest.

## Publisher's note

All claims expressed in this article are solely those of the authors and do not necessarily represent those of their affiliated organizations, or those of the publisher, the editors and the reviewers. Any product that may be evaluated in this article, or claim that may be made by its manufacturer, is not guaranteed or endorsed by the publisher.

## Author disclaimer

The statements made and views expressed are solely the responsibility of the Fellow. For the purpose of open access, the author has applied a CC BY public copyright licence to any Author Accepted Manuscript version arising from this submission.
